# A Revision of the *Prionus gahani* Species Group (Coleoptera: Cerambycidae: Prioninae: Prionini)

**DOI:** 10.3390/insects17040417

**Published:** 2026-04-14

**Authors:** Mei-Ying Lin, Alain Drumont

**Affiliations:** 1 Engineering Research Center for Forest and Grassland Disaster Prevention and Reduction/Ecological Security and Protection Key Laboratory of Sichuan Province, School of Life Sciences (School of Ecological Forestry), Mianyang Normal University, 166 Mianxing West Road, Mianyang 621000, China; linmeiying2021@126.com; 2 O.D. Taxonomy and Phylogeny–Entomology, Royal Belgian Institute of Natural Sciences, Vautier Street 29, B-1000 Brussels, Belgium

**Keywords:** new species, longhorn beetle, new distribution record, male genitalia, diurnal lifestyle, taxonomy

## Abstract

This study contributes to the taxonomic research on the “*Prionus gahani*” species group, which includes six species. A new species, *Prionus zhumingyui* Lin & Drumont, sp. nov., is described from Guangdong Province, China. The new species is distinguished by having pronotum longer, antennomere III with obvious apical lobe more stretched outward and cone-shaped. Illustrations of the habitus and genitalia of the new species and other four species are provided. The habitats and collecting experiences of four species are provided: *P. zhumingyui* Lin & Drumont, sp. nov., *P. sontinh* Do, Drumont & Komiya, 2019 (with new locality information), *P. antonkozlovi* Drumont & Komiya, 2021, and *P*. *gahani* Lameere, 1912 (with female described for the first time). It is concluded that the species of this group are diurnal, although they have somber colors. Their small compound eyes (especially compared with other *Prionus* species, which have large compound eyes and are attracted to light) are well-matched to their diurnal lifestyle.

## Simple Summary

A new species of the genus *Prionus* Geoffroy, 1762, *P. zhumingyui* Lin & Drumont, sp. nov., is described from Guangdong Province, China. This species belongs to the “*Prionus gahani*” species group. Based on collecting experiences of four species, habitat pictures and some biological information are provided. It is concluded that the species of this group are diurnal, although they have somber colors.

## 1. Introduction

*Prionus* Geoffroy, 1762 is a genus of the subfamily Prioninae, tribe Prionini, with *Cerambyx coriarius* Linnaeus, 1758 as type species [1,2]. The known members of *Prionus* are currently widespread in Europe, Asia, and North America [3], though species from some countries of Asia should belong to different genera or subgenera [2]. Before this study, there were 39 species and subspecies in *Prionus* (*s. str.*) in total [4], with 17 taxa (species and subspecies) distributed in China [5,6]. The “*Prionus gahani*” species group was first proposed by Do et al. (2019), who included four species “characterized in having distance between the upper eye lobes much longer than the width of the upper eye lobe, and the antennae usually much shorter than four-fifths of the body length” [3]. Later, Drumont & Komiya (2021) added a fifth species, *P. antonkozlovi* from Fujian Province, China, and mentioned *P. sontinh* from Yunnan Province, also from China [6]. The “*Prionus gahani*” species group currently comprises five valid species distributed in China and Vietnam.

During the examination of material collected in Guangdong Province, China, we identified one new species belonging to the “*Prionus gahani*” species group, herein described as *Prionus zhumingyui* sp. nov. after the collector Mr. Ming-Yu Zhu. By gathering the collected experiences of four species, the biological information of the species in this group is discussed.

## 2. Materials and Methods

The male genitalia were prepared by extracting them with forceps from fresh specimens without removing the abdomen. They were then cleared in 10% KOH at room temperature for 24 h, subsequently transferred to distilled water for rinsing, and examined for observation. Genitalia were photographed submerged in ethyl alcohol and subsequently preserved in polyethylene genitalia vials filled with glycerin, and pinned under the specimens.

Habitus images were taken using a Canon EOS 7D camera with a Canon Macro 100 mm macro lens, Canon, Tokyo, Japan. Images of the same object at different focal planes were combined using Helicon Focus 8 stacking software. Adobe Photoshop CS6 was used for postprocessing. The terminalia were photographed with a Keyence VHX-1000C large-depth-of-field 3D digital microscope, Keyence, Osaka, Japan [7].

The specimens studied are deposited in the following institutional museums and private collections; abbreviations as shown in the text:

**ADC**—Collection of Alain Drumont, Bruxelles, Belgium;

**BMNH**—Natural History Museum, London, United Kingdom;

**CGL**—Collection of Liang Guo, Sanming, Fujian, China;

**CLTK**—Collection of Tao-Kun Liao, Sanming, Fujian, China;

**CZBC**—Collection of Chengzhi Bian, Shandong, China;

**IZCAS**—Institute of Zoology, Chinese Academy of Sciences [=NACRC National Animal Collection Resource Center], Beijing, China;

**MNHN**—Muséum national d’Histoire naturelle, Paris, France;

**MYNU**—Invertebrate collection of Mianyang Normal University, Mianyang, Sichuan, China;

**RBINS**—Royal Belgian Institute of Natural Sciences, Brussels, Belgium;

**ZMUM**—Zoologisk Museum, University of Copenhagen, Denmark.

Abbreviations used: TL: Type locality, TD: Type depository.

This article is registered in ZooBank under the following address:

http://zoobank.org/urn:lsid:zoobank.org:pub:24458DD1-986D-4961-8369-8DDCD0035D31

## 3. Results

### 3.1. Prionus zhumingyui *Lin & Drumont, sp. nov.*

http://zoobank.org/urn:lsid:zoobank.org:act:CB3D0815-860F-4E1D-84CE-8C82D8F54C8A

**Figure 1:**
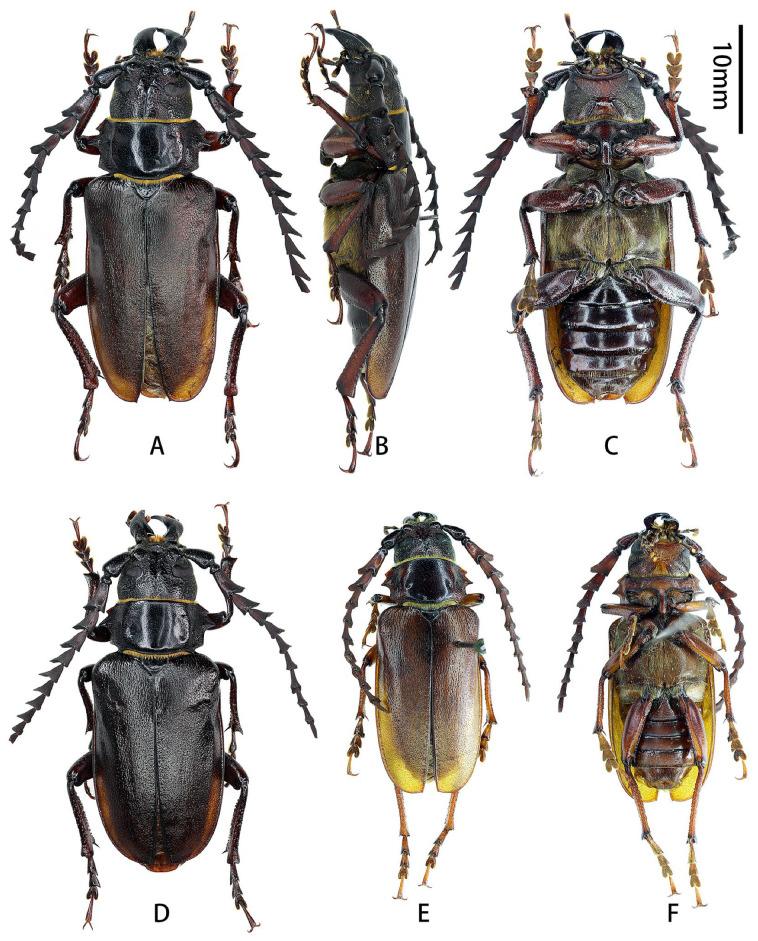
Habitus of *Prionus zhumingyui* Lin & Drumont, **sp. nov.** (**A**–**C**) Holotype male, from Guangdong. (**D**–**F**) Paratypes males, from Guangdong. (**A**,**D**,**E**) Dorsal views. (**B**) Lateral views. (**C**,**F**) Ventral views. Scale bar: 10 mm.

**Figure 2:**
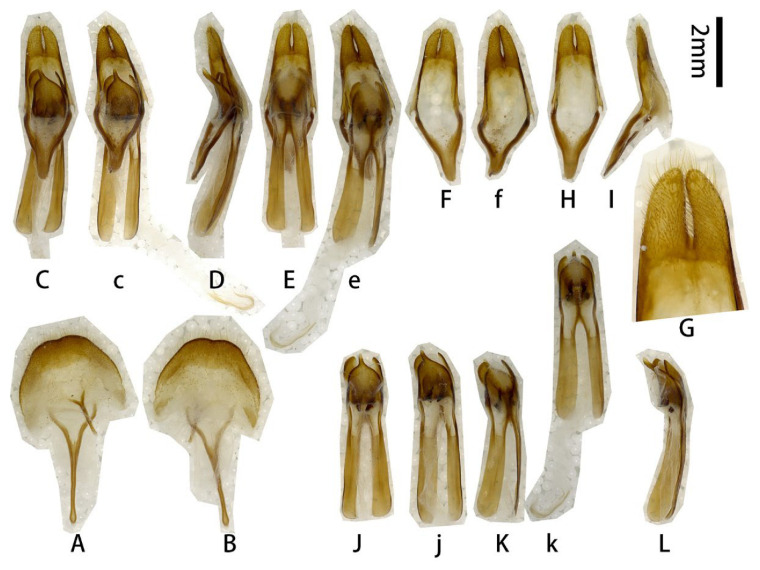
Terminalia of *Prionus zhumingyui* Lin & Drumont, **sp. nov**. (**A**–**L**) Male, holotype. (**c**,**e**,**f**,**j**,**k**) Male, paratype. (**A**,**B**) Tergite VIII with sternites VIII and IX. (**C**–**E**) Male genitalia, internal sac incomplete. (**c**,**e**) Male genitalia, internal sac complete. (**F**–**I**,**f**) Tegmen. (**J**–**L**,**j**,**k**) Median lobe. (**A**,**C**,**c**,**F**,**f**,**G**,**J**,**j**) Ventral views. (**B**,**E**,**e**,**H**,**K**,**k**) Dorsal views. (**D**,**I**,**L**) Lateral views. Scale bar: 2 mm. G not to scale.

**Figure 3:**
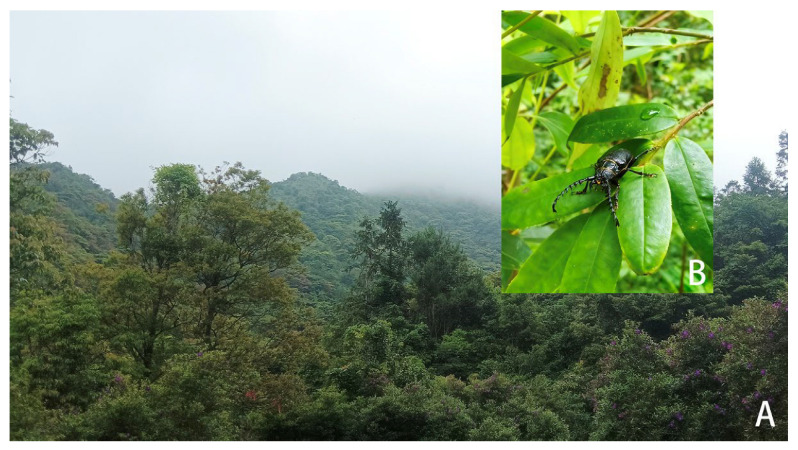
*Prionus zhumingyui* Lin & Drumont, **sp. nov.**, in Guangdong, Maoming City, Xinyishi, Mt. Datianding, photographed by Bao-Zhen Xu. (**A**) The environment of the type locality. (**B**) A living specimen on leaves of *Eurya* sp. B located in the lower left corner of A.

Figure 1A–F, Figure 2A–L,c,e,f,j,k, and Figure 3A,B.

Chinese common name: 朱氏锯天牛.

**Description (male):** Body length: 26.0–33.5 mm, humeral width 9.5–11.5 mm. Body moderately small, stout, reddish brown to blackish brown, dorsoventrally flattened.

Head shorter than wide, remarkably punctured all over; jugular process short, triangularly pointed apically. Eyes small, narrow, separated distantly; distance between upper eye lobes about half width of head, much longer than the width of the upper eye lobe; distance between lower eye lobes 5/8 of width of head. Labrum covered with long setae. Mandibles punctate, robust, acute at apex, slightly bent inwards, with external surface rather strongly bent (curved) at middle, furnished with distinct but not acute inner tooth near base. Antennae with 12 antennomeres, short, about two-thirds of body length. Scape short, robust, narrow basally, strongly expanded to apex, bent inward just after base. Antennomere II about one-fourth as long as scape. Antennomere III slightly shorter than antennomeres I and II combined, dorsoventrally flattened, narrow at base, and expanded apically. Antennomere IV about two-thirds as long as III, flattened, thicker than V; antennomeres V to XI similar to IV, gradually reducing in basal width while gradually longer for apical lobes; antennomere V as long as IV, slightly longer than VI; antennomeres VI–XI almost equal in length; antennomeres IV–XI nearly triangular in dorsal view, acutely pointed apically; antennomere XII irregularly truncate apically; basal area of antennomeres IV–XII strongly narrowed. Ventral side of head flattened and granulate apically.

Prothorax long when compared to allied species (*Prionus gahani* Lameere, 1912 [8], *P. lameerei* Semenov, 1927 [9], *P. sifanicus* Plavilstshikov, 1934 [10]), about one half of as long as wide (measured between tips of middle spines), deeply punctuate, convex on the disk, flattened laterally; each flattened area about one-fifth of pronotal width; with two spines at each side curved backward; anterior spine smaller than middle one; posterior angles almost forming a right angle; hind edge slightly roundly expanding posteriorly; anterior and posterior edges fringed with row of long golden setae.

Scutellum (Figure 1A,D,E) transverse, about twice as broad as long, glabrous.

Elytra flattened, short, about 1.7 times as long as wide; widest area at basal ninth, gradually, slightly narrowed toward base, nearly parallel-sided from basal ninth to apical fifth, and rounded apically; sutural spine short.

Legs flattened, robust. Femora smooth, thick, about as long as tibiae, ventral side with deep groove which is widened from base to apex (Figure 1C,F). Tibiae granulate, sinuous, curved outward distally, with two tibial spurs; inner side with deep groove, which is widened from base to apex. Tarsi with similar shape, combined length of tarsomeres I–V about as long as tibia; tarsomere V longest, tarsomere I subequal to (metatarsus) or slightly shorter (pro- and mesotarsi) than tarsomere V; tarsomeres II and III with similar length.

**Male genitalia** (Figure 2A–L): Tergite VIII (Figure 2B) transverse; apex slightly emarginated in middle; setae short and moderately dense. Tegmen rhombic in ventral view (Figure 2F,f), moderately curved in lateral view (Figure 2I); parameres (Figure 2f,G) narrower apically, moderately slender (length/width more than 2.5) with rounded apex, entirely covered with dense short setae. Median lobe very slightly curved (Figure 2L) in lateral view, median struts more than half length of median lobe. Ventral plate of median lobe strongly projected (Figure 2J,j), dorsal plate of median lobe with two wide lobes (Figure 2K,k). Internal sac with two pieces of crest-shaped basal armature (Figure 2J–L), with a bank of darker colored area at apex (Figure 2c,e,k).

Female unknown.

**Figure 4:**
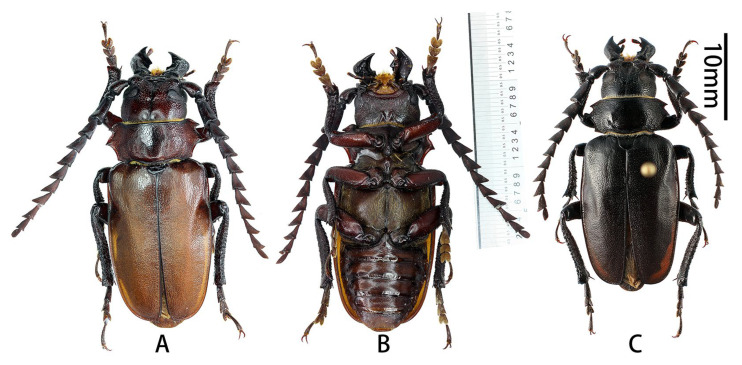
Habitus of *Prionus sontinh* Do, Drumont & Komiya, 2019. (**A**,**B**) male, from Hà Giang, Vietnam. (**C**) male, from Yunnan. (**A**,**C**) Dorsal views. (**B**) Ventral views. Scale bar: 10 mm.

**Diagnosis:** Among the five species composing the “*Prionus gahani*” species group [3], *Prionus zhumingyui* **sp. nov.** is closest to *P. sontinh* (Figure 4A–C) but can be easily distinguished by having pronotum longer (ca. 1/2 vs. 1/3 as long as wide), antennomere III with obvious apical lobe more stretched outward and cone-shaped, parameres slender, and tergite VIII with apex slightly emarginated in middle.

**Type material. HOLOTYPE: CHINA**: ♂ (Figure 1A–C and Figure 2A–L), Guangdong, Maoming City, Xinyishi, Mt. Datianding (广东茂名市信宜市大田顶山, Figure 3A), alt. ca. 1560 m, 22.V.2025, leg. Ming-Yu Zhu (IZCAS). **PARATYPES: CHINA:** 1 ♂ (Figure 1D and Figure 2c,e,f,j,k), same data as holotype but deposited in MYNU (molecular number ZH11); 1♂ (Figure 1E,F), Guangdong, Xinyishi, Datianding (广东信宜大田顶), alt. 1704 m, 2.VI.1984, leg. Su-Bai Liao (IZCAS).

**Distribution:** China (Guangdong). So far only known from Mt. Datianding; more investigations in this area will contribute to better define the distribution of *Prionus zhumingyui* Lin & Drumont, **sp. nov**.

**Etymology:** The new species is dedicated to the local collector Mr. Ming-Yu Zhu (Conghua District, Guangzhou, Guangdong), who donated two type specimens for this study and many other longhorned beetles to the first author. The name is a noun in the genitive case.

**Biology and Ecology:** According to Mr. Ming-Yu Zhu and Mr. Bao-Zhen Xu, the beetle was resting on leaves of *Eurya* sp. (Pentaphylacaceae) (Figure 3B) at 16: 30, and the weather was sunny. The habitat is located at an altitude of 1560 m in forests usually covered by fog, with very high humidity (Figure 3A). The beetle was occasionally resting on leaves of *Eurya* sp.; therefore, the host plant is still unknown. The specimens were collected from 22 May to 2 June, so May and June should be their active adult period. No individual came to light trap setting in the middle of this mountain, which indicated that it is active during the day, not nocturnal.

### 3.2. Prionus sontinh *Do, Drumont & Komiya, 2019*

**Figure 5:**
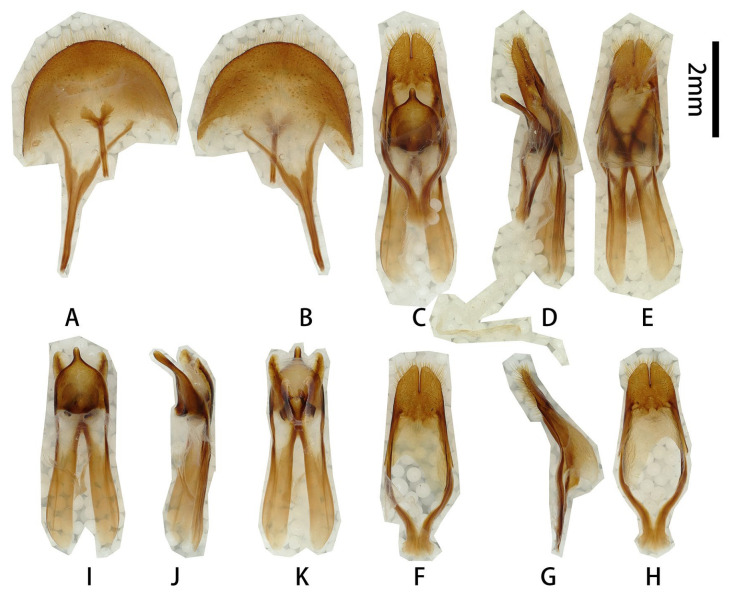
Terminalia of *Prionus sontinh* Do, Drumont & Komiya, 2019. (**A**,**B**) Tergite VIII with sternites VIII and IX. (**C**–**E**) Male genitalia. (**F**–**H**) Tegmen. (**I**–**K**) Median lobe. (**A**,**C**,**F**,**I**) Ventral views. (**B**,**E**,**H**,**K**) Dorsal views. (**D**,**G**,**J**) Lateral views. Scale bar: 2 mm.

Figure 4A–C and Figure 5A–K.

Chinese common name: 山神锯天牛.

*Prionus sontinh* Do, Drumont & Komiya, 2019: 63, Figures 1–8 [3]. Type locality: Vietnam: Lai Chau, Tam Duong.

*Prionus sontinh*: Drumont & Komiya, 2021: 130, Figure 5 [6].

**Examined material**: Holotype, ♂, Vietnam, Lai Châu, Tam Duong, VI.2016, leg. Cuong Do (RBINS).

**Vietnam:** 1♂ (Figure 4A,B), Hà Giang, V.2025 (CZBC, molecular number SO14); 3♂♂, Hà Giang, Xin Man, VI.2022 (ADC; one male will be deposited in RBINS, I.G.: 35.082)

**China:** 3♂♂ (Figure 4C), Yunnan, Pingbian, Mt. Daweishan (云南屏边大围山), 2000 m, IV–V. 2020 (ADC; one male will be deposited in RBINS, I.G.: 35.082); 1♂, Yunnan, Jinping, Fenshuiling (云南金平分水岭), VII.2023 (ADC).

**Complementary description.** Male body length 24.0–38.5 mm, humeral width 10.0–15.0 mm; female body length 23.0–32.0 mm, humeral width 12.0–14.0 mm.

Based on the newly examined specimens during this study, the male specimens from Hà Giang, Vietnam, are reddish brown, while the male specimens from Yunnan, China, are shining black. These variations are observed and reported from the type series too [3].

**Male genitalia** (Figure 5A–K): Tergite VIII (Figure 5B) transverse, apex broadly rounded, setae short and moderately dense. Tegmen rhombic in ventral view (Figure 5F), slightly curved in lateral view (Figure 5G); parameres (Figure 5F) narrower apically, quite stout (length/width ca. 1.4) with obliquely rounded apex, entirely covered with dense and moderately long setae. Median lobe very slightly curved (Figure 5J) in lateral view, with median struts about 4/7 of median lobe length. Ventral plate of median lobe strongly projected (Figure 5I), dorsal plate of median lobe with two narrow lobes (Figure 5K). Internal sac with two pieces of crest-shaped basal armature (Figure 5I–K).

**Distribution:** China (Yunnan) and Vietnam. This species was described from Lai Châu Province, northern Vietnam [3]. Yunnan was reported by Drumont & Komiya (2021) without detailed data [6], which are reported here for the first time. The species has also recently been found in the Vietnamese province of Hà Giang (Vi Xuyên and Xin Man), which shares more than 200 km of boundary with the province of Yunnan.

**Biology and Ecology:** According to Mr. Cheng-Zhi Bian, the *Prionus sontinh* specimens living on the Mt. Daweishan were active during the day and were most commonly found on cement roads in mountains. They have not been observed to fly, and males are not coming to light traps. Specimens live at a relatively high altitude, around 1500–2000 m, and the habitat is represented by virgin forests, with no artificial or secondary forests present. Rotting trunks were examined for individuals with no success.

### 3.3. Prionus antonkozlovi *Drumont & Komiya, 2021*

**Figure 6:**
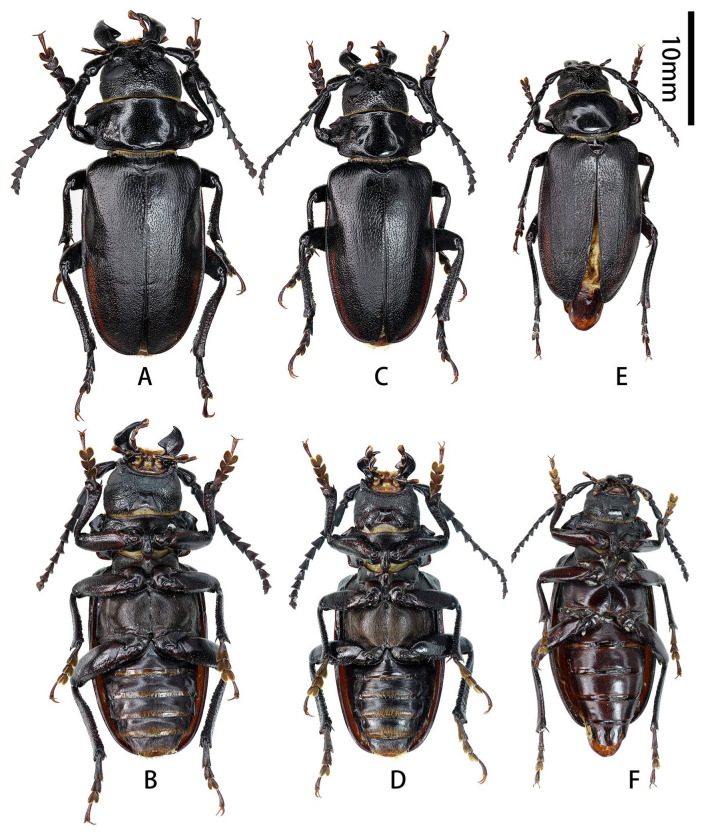
Habitus of *Prionus antonkozlovi* Drumont & Komiya, 2021. (**A**–**D**) Males, from Fujian. (**E**,**F**) Female, from Fujian. (**A**,**C**,**E**) Dorsal views. (**B**,**D**,**F**) Ventral views. Scale bar: 10 mm.

**Figure 7:**
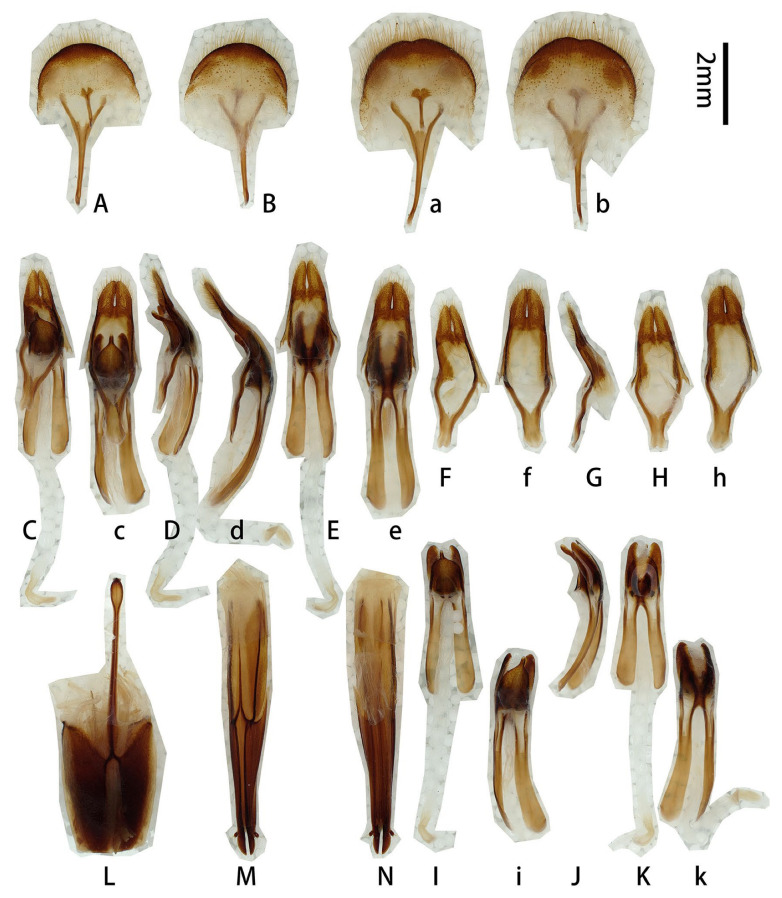
Terminalia of *Prionus antonkozlovi* Drumont & Komiya, 2021 from Fujian. (**A**–**K**) Male, smaller. (**a**–**f**,**h**,**i**,**k**) Male, larger. (**L**–**N**) Female. (**A**,**B**,**a**,**b**) Tergite VIII with sternites VIII and IX. (**C**–**E**) Male genitalia, internal sac complete. (**c**–**e**) Male genitalia, internal sac incomplete. (**F**–**H**,**f**,**h**) Tegmen. (**I**–**K**,**i**,**k**) Median lobe. (**L**) Female, sternite VIII with spiculum ventral (also called tignum). (**M**,**N**) Female, ovipositor. (**A**,**a**,**C**,**c**,**F,f**,**I**,**i**,**L**,**M**) Ventral views. (**B**,**b**,**E**,**e**,**H**,**h**,**K**,**k**,**N**) Dorsal views. (**D**,**d**,**G**,**J**) Lateral views. Scale bars: 2 mm.

**Figure 8:**
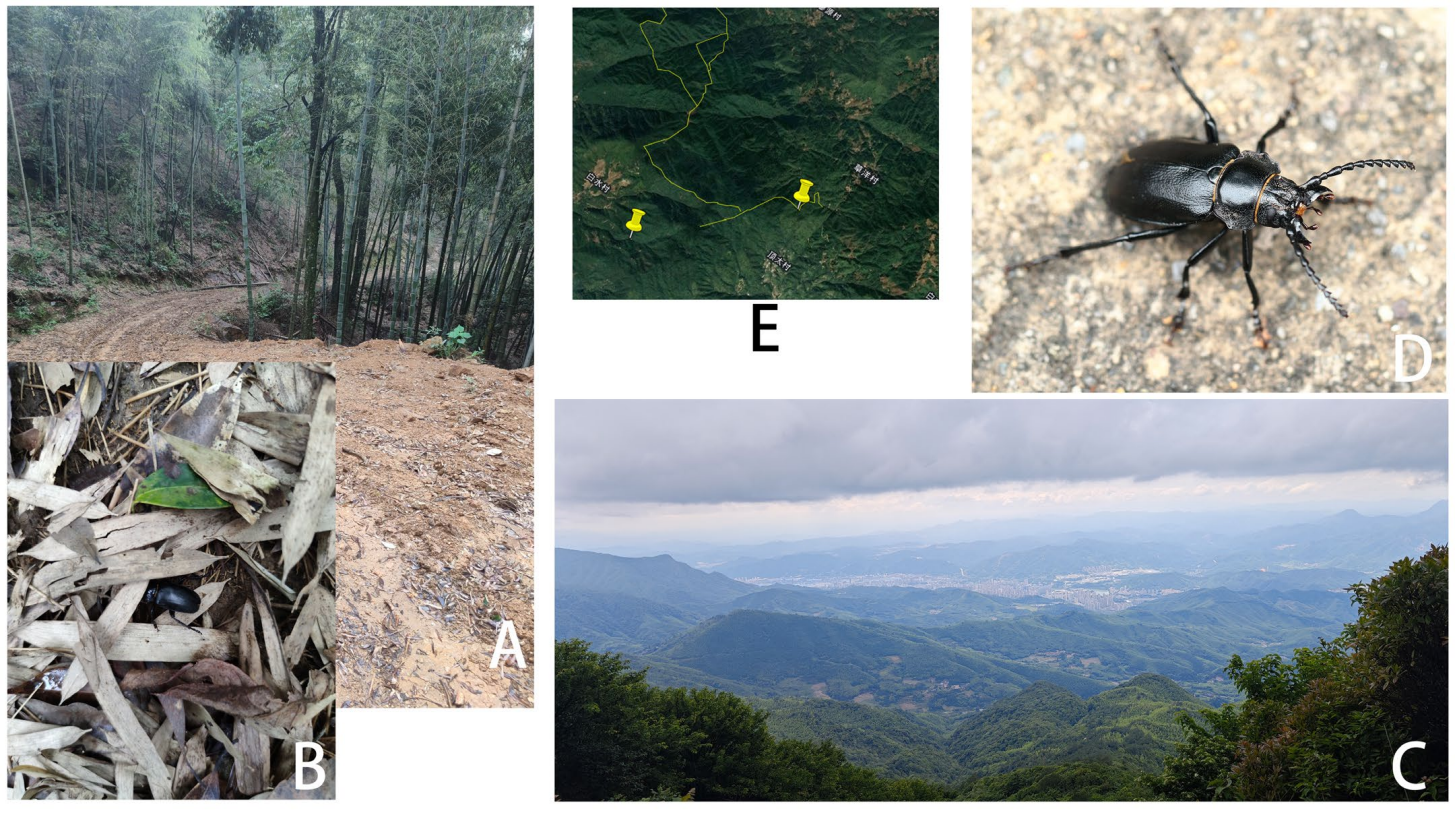
*Prionus antonkozlovi* Drumont & Komiya, 2021, in Fujian, Sanming City. (**A**) The environment of Sanyuan Distr., Lianhuafeng. (**B**) A living specimen under dry leaves of bamboo. B is located in the bamboo forest of A. (**C**) The environment of type locality, Shaxian, Luobading. (**D**) A living specimen crawling on a road of the type locality. (**E**) Collecting sites: the left yellow point is Lianhuafeng; the right yellow point is the type locality, Luobading. The straight-line distance between the two points is three kilometers, the three villages’ names in Chinese from left to right are Baishuicun (白水村), Dingtaicun (顶太村) and Caoyangcun (草洋村). All photographed by Tao-Kun Liao except D photographed by Liang Guo.

Figure 6A–F, Figure 7A–N,a–f,h,i,k, and Figure 8A–E.

Chinese common name: 科兹洛夫锯天牛.

*Prionus antonkozlovi* Drumont & Komiya, 2021: 127, Figure 1 [6]. Type locality: China: Fujian, Sanming, Luobading.

**Complementary description.** Male body length 22.0–35.0 mm, humeral width 8.0–13.0 mm; female body length 23.0–34.0 mm, humeral width 8.5–13.5 mm.

**Male genitalia** (Figure 7A–K,a–f,h,i,k): Tergite VIII (Figure 7B) transverse, apex broadly rounded (slightly emarginated in another larger male, Figure 7a,b), setae short and moderately dense. Tegmen rhombic in ventral view (Figure 7F,f), slightly curved in lateral view (Figure 7G); parameres (Figure 7F,f) narrower apically, moderately stout (length/width ca. 2.0) with rounded apex, entirely covered with dense short setae. Median lobe slightly curved (Figure 7D,d,J) in lateral view, median struts about 3/5 of median lobe length. Ventral plate of median lobe strongly projected (Figure 7I,i), dorsal plate of median lobe with two wide lobes (Figure 7K,k). Internal sac with two pieces of crest-shaped basal armature (Figure 7I–K). **Female** sternite VIII with spiculum ventral (also called tignum) and ovipositor as in Figure 7L–N.

**Examined material:** Holotype, ♂, Fujian, Sanming, Luobading (福建三明市锣钹顶), VI.2015 (RBINS, I.G.: 34.305); Allotype, 1♀, same data as holotype.

**China, Fujian:** 1♂, Fujian, Sanming City, Shaxian, Luobading (Figure 8C, 福建三明市沙县锣钹顶), elev. 1100 m, 18.VI.2019, leg. Liang Guo (CGL); 2♂♂ (Figure 6A–D), Fujian, Sanming City, Shaxian, Mt. Luobading (福建三明市沙县锣钹顶), elev. 1300 m, 30.V.2025, leg. Tao-Kun Liao (CLTK, molecular number AN9); 3♂♂1♀, Fujian, Sanming City, Shaxian, Mt. Luobading (福建三明市沙县锣钹顶), elev. 1300 m, 2.VI.2025, leg. Tao-Kun Liao (CLTK); 1♂1♀ (Figure 6E,F), Fujian, Sanming, Sanyuan Distr., Lianhuafeng (Figure 8A, 福建三明市三元区莲花峰), elev. 1100 m, 1.VI.2021, leg. Tao-Kun Liao (CLTK, the female has 219 eggs for molecular study, AN10); 2♀♀, same data but elev. 1200 m, 9.VI.2023; 1♂1♀, same data but VII.2016, leg. Liang Guo (CGL); 1♂, Fujian, Sanming, Yong’anshi, Zhushekeng (福建三明市永安市竹畲坑), elev. 1100 m, 18.VI. 2019, leg. Tao-Kun Liao (CLTK).

**Distribution:** China (Fujian).

**Biology and Ecology:** According to Mr. Tao-Kun Liao, Mr. Liang Guo, and Mr. Peng-Yu Liu, this beetle occasionally appears in drainage ditches or crawls on the ground at the roadside, and is not attracted to light. According to field observations by Mr. Liang Guo and Mr. Tao-Kun Liao, this beetle just disappears at night, does not crawl on the ground, and cannot be attracted to light. This species has been collected in some areas of the Daimao Mountain Range (including Lotus Peak, Puchan Mountain, etc.), South of Sanming City, Fujian. It is mainly distributed at altitudes between 800 and 1400 m, with 1100 m being the primary elevation range. Its habitat includes both bamboo forests and beech forests. The peak collection period is from May to June, when the beetles are particularly active, crawling on the ground after rain.

As an additional biological information, 219 eggs were found in a female with a 23 mm length.

### 3.4. Prionus lameerei *Semenov, 1927*

Figure 9A.

Chinese common name: 云南锯天牛.

*Prionus gahani*: Lameere, 1916: 257 (misidentification) [11].

*Prionus lameerei* Semenov, 1927: 235 [9]. Type locality: China: Yunnan.

*Prionus lameerei*: Gressitt, 1951: 24, 26 [12]; Wang, 1986: 48 [13]; Hua, 2002: 226 (catalogue) [14]; Löbl & Smetana, 2010: 94 (catalogue) [15]; Li et al., 2014: 92 [16]; Chen et al., 2019: 23 (catalogue) [5]; Danilevsky, 2020: 116 (catalogue) [17]; Drumont & Komiya, 2021: 130, Figure 3 [6].

*Prionus lameeri*: Wang & Chiang, 1988: 146 (misspelling) [18].

**Figure 9:**
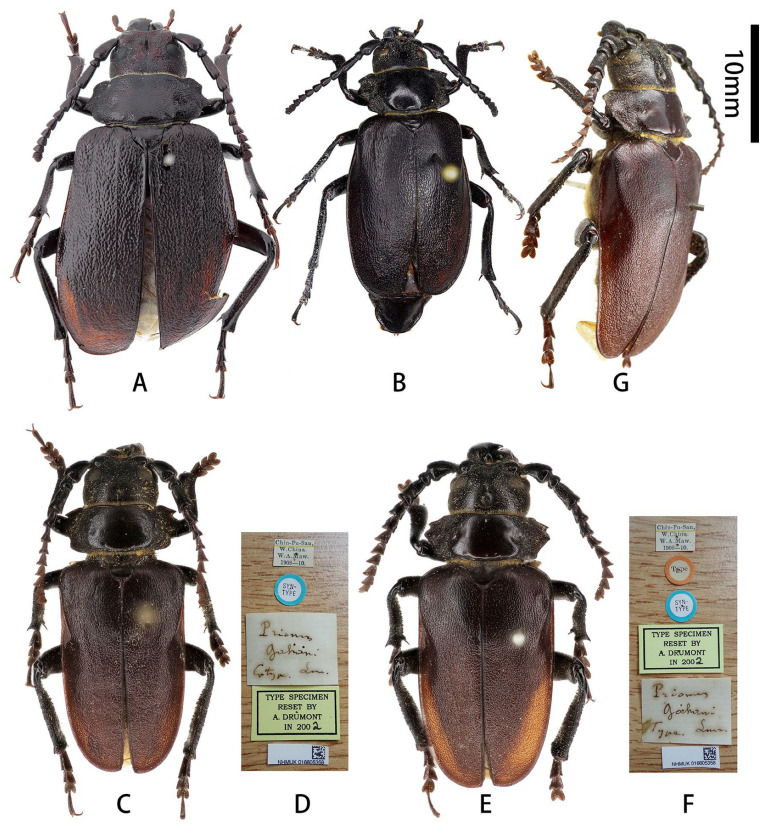
Habitus of *Prionus* spp. (**A**) *Prionus lameerei* Semenov, 1927, holotype female, from Yunnan. (**B**–**G**) *Prionus gahani* Lameere, 1912. (**B**) Female, from Sichuan. (**C**,**D**) Lectotype male, from Chongqing. (**E**–**G**) Paralectotype male, from Chongqing. (**A**–**C**,**E**) Dorsal views. (**G**) Lateral views. Scale bar: 10 mm. (**D**,**F**) Labels. Not to scale.

**Examined material:** Holotype (Figure 9A), ♀, Yunnan (云南), leg. P. Guerry (MNHN).

**Distribution:** China (Yunnan, Sichuan?).

**Remarks.** The record from Sichuan by Wang (1986) was doubtful; it might be a misidentification of *Prionus gahani*.

### 3.5. Prionus gahani *Lameere, 1912*

**Figure 10:**
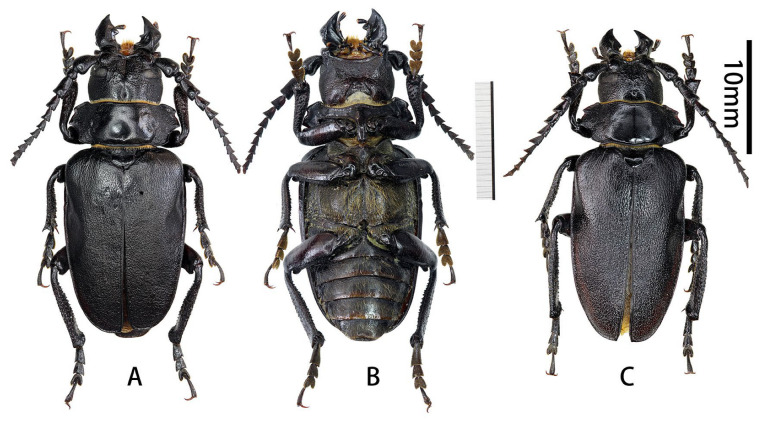
Habitus of *Prionus gahani* Lameere, 1912, from type locality Chongqing, Jinfoshan. (**A**,**C**) Dorsal views. (**B**) Ventral view. Scale bar: 10 mm.

**Figure 11:**
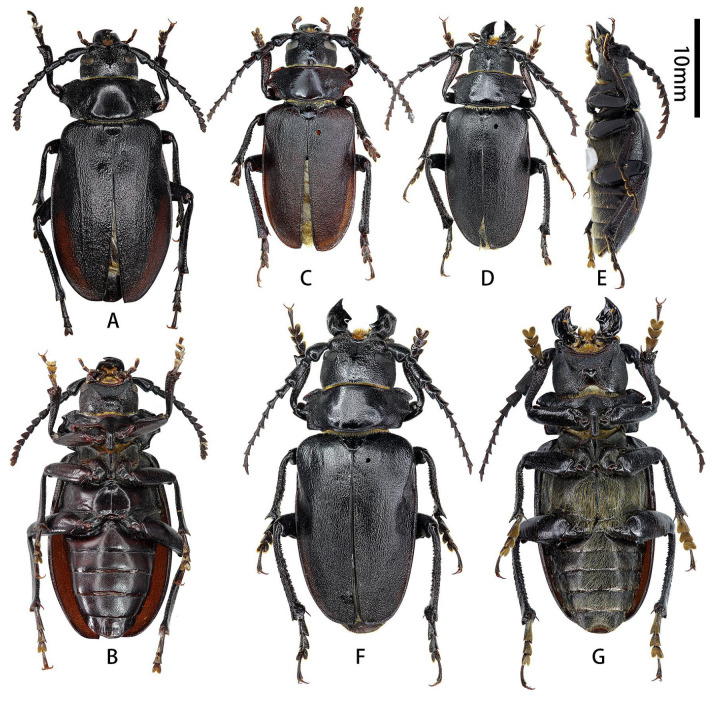
Habitus of *Prionus gahani* Lameere, 1912. (**A**,**B**) Female from Chongqing, Wanxian. (**C**) Male from Chongqing, Wulong. (**D**–**G**) Males from Sichuan. (**A**,**C**,**D**,**F**) Dorsal views. (**E**) Lateral view. (**B**,**G**) Ventral views. Scale bar: 10 mm.

**Figure 12:**
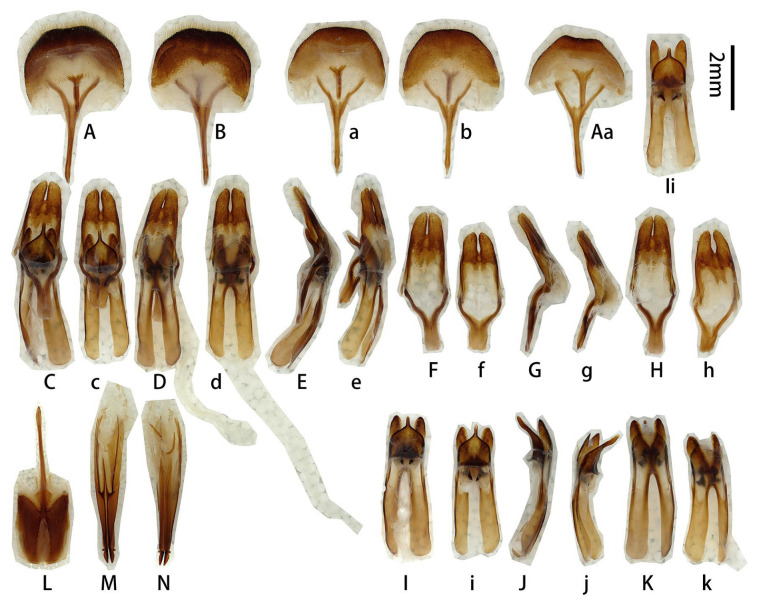
Terminalia of *Prionus gahani* Lameere, 1912, from Chongqing. (**A**–**K**) Male, specimen Figure 10A,B. (**a**–**k**) Male, specimen Figure 10C. (**L**–**N**) Female, specimen Figure 11A,B. (**Aa**, **Ii**) Male, from Chongqing. (**A**,**B**,**a**,**b**,**Aa**) Tergite VIII with sternites VIII and IX. (**C**–**E**,**c**–**e**) Male genitalia. (**F**–**H**,**f**–**h**) Tegmen. (**I**–**K**,**i**–**k**,**Ii**) Median lobe. (**L**) Female, sternite VIII with spiculum ventral (also called tignum). (**M**,**N**) Female, ovipositor. (**A**,**a**,**Aa**,**C**,**c**,**F**,**f**,**I**,**i**,**Ii**,**L**,**M**) Ventral views. (**B**,**b**,**D**,**d**,**H**,**h**,**K**,**k**,**N**) Dorsal views. (**E**,**e**,**G**,**g**,**J**,**j**) Lateral views. Scale bar: 2 mm.

**Figure 13:**
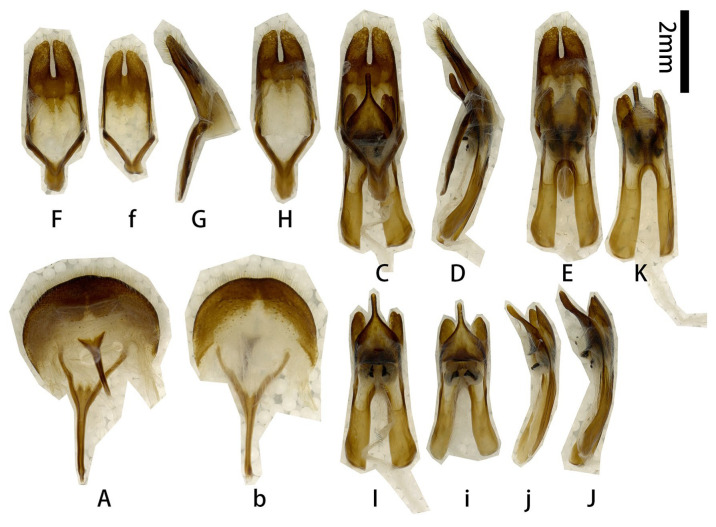
Terminalia of *Prionus gahani* Lameere, 1912, from Sichuan. (**A,C**–**K**) Male, bigger size. (**b**,**f**,**i**,**j**) Male, smaller size. (**A**,**b**) Tergite VIII with sternites VIII and IX. (**C**–**E**) Male genitalia, internal sac incomplete. (**F**–**H**,**f**) Tegmen. (**I**–**K**,**i**,**j**) Median lobe. (**A**,**C**,**F**,**f**,**I**,**i**) Ventral views. (**b**,**E**,**H**,**K**) Dorsal views. (**D**,**G**,**J**,**j**) Lateral views. Scale bar: 2 mm.

**Figure 14:**
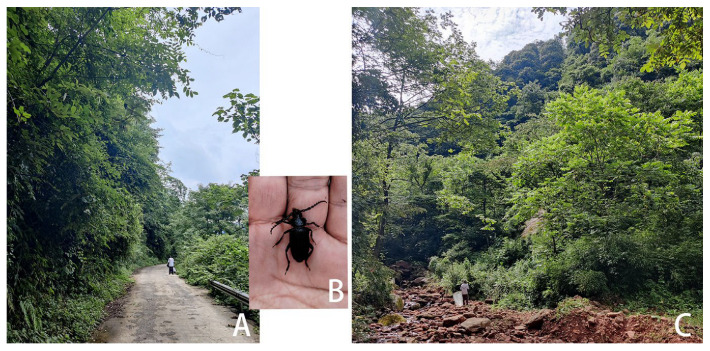
*Prionus gahani* Lameere, 1912 in Sichuan, Luzhou City, Gulin County. (**A**) Habitat in Huangjing Town, photographed by Hao Xu. (**B**) A living specimen collected by sweeping net on the road of Figure A, photographed by Can Zhang. (**C**) Habitat in Zhangde Town, photographed by Hao Xu.

Figure 9B–G, Figure 10A–C, Figure 11A–G, Figure 12A–N,a–k,Aa,Ii, Figure 13A,C–K,b,f,i,j and Figure 14A–C.

Chinese common name: 短角锯天牛.

*Prionus gahani* Lameere, 1912: 189 [8]. Type locality: China: Chongqing (Chin-Fu-San).

*Prionus gahani*: Lameere, 1919: 132, pl. 6, Figure 5 ([19], part); Gressitt, 1951: 24, 25 [12]; Hua, 2002: 226 (catalogue) [14]; Löbl & Smetana, 2010: 94 (catalogue) [15]; Li et al., 2014: 91 [16]; Chen et al., 2019: 22 (catalogue) ([5], part, wrong type locality); Danilevsky, 2020: 116 (catalogue) [17]; Drumont & Komiya, 2021: 130, Figure 2 [6].

**Examined material:** Lectotype ♂ (Figure 9C,D), Chin-Fu-San, W. China, 1908-10, W. A. Maw (BMNH, NHMUK 016605359); paralectotype ♂ (Figure 9E–G), Chin-Fu-San, W. China, 1908-10, W. A. Maw (BMNH, NHMUK 016605358).

**Lectotype designation:**

According to Lameere’s original description, there was more than one syntype. We therefore designate as the male lectotype (Figure 9C,D) (ICZN, Recommendation 73 F No presumption of the existence of a holotype) the specimen held at the BMNH and bearing the following labels (Figure 9D):

(1) rectangular, white, printed in black with a yellow line “Chin-Fu-San, W. China, 1908–10, W. A. Maw”,
(2) round, white, with a blue border, printed in black “SYNTYPE”,
(3) rectangular, white, handwritten by Lameere “Prionus gahani Cotype Lmr.”,
(4) yellow, printed with a black border: “Type specimen reset by A. DRUMONT in 2002”,
(5) rectangular, white, with a QR code and printed in black: NHMUK 016605359.

The lectotype is in very good condition; only the last tarsomere of right pro- and mesotarsi, as well as the claws of the right metatarsus, are missing.

We designate as paralectotype male (Figure 9E–G) the specimen held at the BMNH and bearing the following labels (Figure 9F):

(1) rectangular, white, printed in black with a yellow line “Chin-Fu-San, W. China, 1908–10, W. A. Maw”,
(2) round, white, with a red border, printed in black “TYPE”,
(3) round, white, with a blue border, printed in black “SYNTYPE”,
(4) rectangular, white, handwritten by Lameere “Prionus gahani Type Lmr.”,
(5) yellow, printed with a black border: “Type specimen reset by A. DRUMONT in 2002”,
(6) rectangular, white, with a QR code and printed in black: NHMUK 016605358.

The lectotype (Figure 9C,D) was selected since its pronotum shows the normal pattern of *Prionus gahani* and is easily observed in males of this species, whereas the paralectotype exhibits a rather atypical pronotum.

**China, Chongqing:** 1♂, Chongqing, Nanchuan, Jinfoshan, Huangniya (重庆南川金佛山黄泥垭, Figure 10A,B and Figure 12A–K), 1350 m, 19.VI.2025, leg. Tian-Xuan Gu, Yong Zhou (MYNU ex Tian-Xuan Gu’s private collection, molecular data GS2); 1♂ (Figure 10C and Figure 12a–k), same data but IZCAS ex Xiao-Tian Zhong’s private collection, molecular data GS1; 1♂ (Figure 12Aa,Ii), Chongqing, Wanxian, Wangerbao (重庆万县王二包), 1200 m, 12.VII.1993, leg. Jian Yao (IZCAS, 8957); 1 ♂, same data but 11.VII.1993 (IZCAS, 11023); 1 ♀ (Figure 11A,B and Figure 12L–N), Chongqing, Wanxian, Wangerbao (重庆万县王二包), 1200 m, 10.VII.1993, leg. Jian Yao by light trap (IZCAS, 10482, molecular data GS5); 2 ♂♂ 1 ♀, same data but (IZCAS, 11023 & 11029 & 11024); 1 ♂, same data but leg. Run-Zhi Huang (IZCAS, 3049); 1 ♂, same data but 10.VII.1993, leg. Run-Zhi Huang (IZCAS, 13256); 1♂, Chongqing, Wanxian, Wangerbao (重庆万县王二包), 1200 m, 9.VII.1993, leg. Wen-Zhu Li (IZCAS, 5157, molecular data GS6); 2 ♂♂, same data but (IZCAS 5159 & 5175); 1 ♂, same data but 10.VII.1993 (IZCAS 126); 1♂ (Figure 11C), Chongqing, Wulong, Baimashan (重庆武隆白马山), 1250 m, 1.VII.1989, leg. Bao-Wen Sun (IZCAS, molecular data GS7).

**China, Sichuan:** 1♀ (Figure 9B), Sichuan, Huaying city, Mt. Huayingshan (四川华蓥市华蓥山), elev. 1400 m, 23 & 24.VI.2007, leg. Wei-Wei Zhang (IZCAS, ex Wei-wei Zhang’s private collection); 1 ♂, Xuanhan County, Nanzhuchang (宣汉楠竹场), 20.VI.1980, leg. Xiao-Hong Chen (IZCAS); 4♂♂ (Figure 11D,E and Figure 13b,f,i,j), Luzhou City, Gulin County, Zhangde Town, Longzhuagonglu, Da’ao (泸州市古蔺县彰德镇龙爪公路大坳, Figure 14C), 1580 m, 28.1782° N, 105.7637° E, 12.VII.2024, leg. Hao Xu, Can Zhang, Yang-Ye Liao (MYNU, molecular data GS2); 3 ♂♂ (Figure 11F,G and Figure 13A–K), Luzhou City, Gulin County, Huangjing Town, Gaofeng village, Liangfeng’ao (泸州市古蔺县黄荆镇高峰村凉风坳), 1000 m, 28.1595° N, 105.6984° E, 15.VII.2024, leg. Hao Xu, Xin-Man Yang, Can Zhang (MYNU); 2 ♂♂, Luzhou City, Gulin County, Huangjing Town, Huangjing village, Changtan (泸州市古蔺县黄荆镇黄荆村长滩), 915 m, 28.2849° N, 105.7741° E, 13.VII.2024, leg. Hao Xu, Xin-Yuan Zhang, Xin-Man Yang (MYNU); 1 ♂ (Figure 14B), Luzhou City, Gulin County, Huangjing Town, Bajiedong village, Shanwangba (泸州市古蔺县黄荆镇八节洞村山王坝, Figure 14A), 1214 m, 28.2263° N, 105.7487° E, 14.VII.2024, leg. Hao Xu, Can Zhang, Yang-Ye Liao (MYNU); 1♂, Luzhou City, Gulin County, Deyao Town, Longmeiguanhuzhan (泸州市古蔺县德耀镇龙美管护站), 1500 m, 28.0901° N, 105.6902° E, 5.VIII.2024, leg. Hao Xu, Xin-Yuan Zhang, Shu-Tong Zhang (MYNU).

**Complementary description.** Male body length 18.0–34.0 mm, humeral width 8.0–14.0 mm; female body length 25.0–32.0 mm, humeral width 10.0–13.0 mm.

**Female described for the first time.** The coloration and punctation (Figure 9B and Figure 11A,B) closely match those of male. Antennae shorter, with last four antennomeres extending beyond basal margin of pronotum; antennomere III cylindrical, slightly shorter than scape; antennomere IV much shorter than III and subequal to V; antennomeres V to XI with short apical lobes; antennomere XII very short and almost rounded at apex. Pronotum with lateral teeth nearly identical to those of male. Elytra distinctly expanded from basal ninth, widest near middle, then gradually narrower posteriorly, with their sutural margins separated from near middle to apex. Each elytron has only one carina near middle, elytral apex identical to that of male. Ventral side (Figure 11B) lacking long pubescence present in males, with ventrite V rounded at apex.

**Male genitalia** (Figure 12A–K,a–k,Aa,Ii and Figure 13A,C–K,b,f,i,j): Tergite VIII (Figure 12B,b and Figure 13A,b) transverse, apex broadly rounded to slightly emarginated, setae short and moderately dense. Tegmen rhombic in ventral view (Figure 12F,f and Figure 13F,f), moderately curved in lateral view (Figure 12G,g and Figure 13G); parameres (Figure 13F) narrower apically, moderately stout (length/width ca. 2.0) with projected apex, entirely covered with dense short setae. Median lobe very slightly curved (Figure 12J,j and Figure 13J,j) in lateral view, median struts subequal to half the length of median lobe. Ventral plate of median lobe strongly projected (Figure 12I,i,Ii and Figure 13I,i), dorsal plate of median lobe with two wide lobes (Figure 12K,k and Figure 13K). Internal sac with two pieces of crest-shaped basal armature (Figure 12I–K,i–k,Ii and Figure 13I–K,i,j). **Female** sternite VIII with spiculum ventral (also called tignum) and ovipositor as in Figure 12L–N.

**Distribution:** China (Gansu, Chongqing, Sichuan).

**Diagnosis of females.** Before this study, *P. lameerei* (known only from the holotype female) was separated from *P. gahani* using male characters. Based on the females examined here, the two species can be distinguished by: (1) punctures overall much denser in *P. gahani* than in *P. lameerei*; (2) antennomeres V to IX with longer apical lobes in *P. gahani* than in *P. lameerei*; (3) lateral pronotal teeth in *P. gahani* with a short but backward-pointing apical tooth and almost right angled base, while in *P. lameerei* the apical tooth is almost absent and the base is broadly rounded; (4) each elytron with only one carina in *P. gahani* but two carinae in *P. lameerei*.

**Biology and Ecology:** According to Dr. Hao Xu, these beetles are particularly active at midday (11:00 to 13:30) on clear, sunny days. While collecting specimens along a forest trail, the collectors (Dr. Hao Xu and his students) observed multiple individuals emerging one after another—and at times, two or three simultaneously—from various spots in the undergrowth (Figure 14C). One male specimen (Figure 14B) was collected in the environment as Figure 14A at the time of 15: 46 on July 14th. Specimens were collected at elevations ranging from 915 to 1580 m. Nighttime light-trapping activities were conducted near the collection sites, but none of these beetles were captured. Once again, this suggests that the species may not be attracted to light.

**Remarks:** The type locality “Chin-Fu-San, W. China” (Figure 9D,F) was mistreated as a place from Yunnan [4,5]. This misunderstanding might be caused by Lameere (1919) [19], who wrote “Nord-Ouest de la Chine, Yunnan”, meaning Nord-Ouest de la Chine “and” Yunnan, but that might be misunderstood as Yunnan “belonging to Nord-Ouest de la Chine”. However, “Chin-Fu-San, W. China” is Jinfoshan, Chongqing, the same as the type locality of *Echinovelleda chinensis* Breuning, 1936 [20,21]. Actually, the “Yunnan” reported by Lameere (1916) [11] and then repeated by Lameere (1919) [19] was based on a misidentified specimen of *Prionus lameerei* [9]. Other catalogues [5,14,15,16,17] just repeated the previous mistake. Therefore, we deleted Yunnan from the distribution list of *P. gahani*.

### 3.6. Prionus sifanicus *Plavilstshikov, 1934*

**Figure 15:**
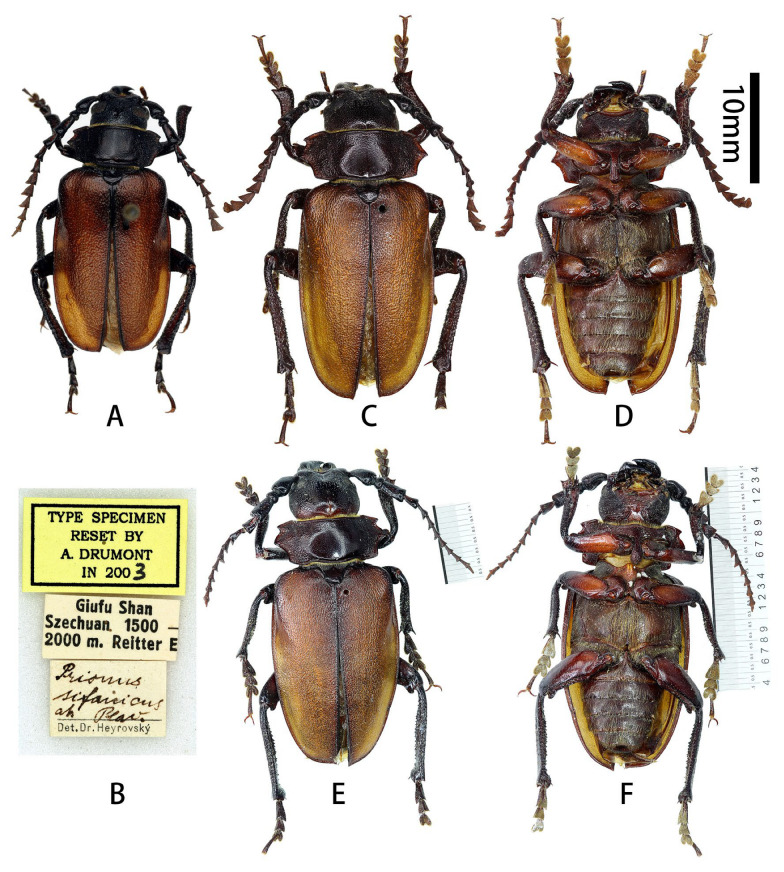
Habitus of *Prionus sifanicus* Plavilstshikov, 1934. (**A**,**B**) Lectotype male from Chongqing, not to scale. (**C**–**F**) Males from Chongqing, Jiangjin. (**A**,**C**,**E**) Dorsal views. (**D**,**F**) Ventral views. Scale bar: 10 mm.

**Figure 16:**
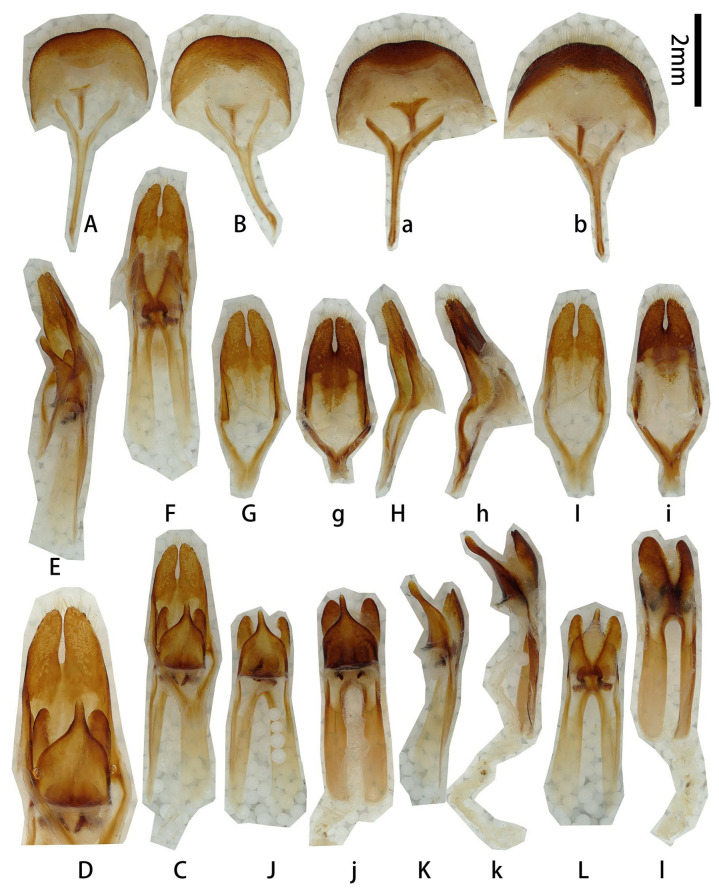
Terminalia of *Prionus sifanicus* Plavilstshikov, 1934. (**A**–**L**) Male, specimen Figure 15C,D. (**a**,**b**,**g**–**l**) Male, specimen Figure 15E,F. (**A**,**B**,**a**,**b**) Tergite VIII with sternites VIII and IX. (**C**–**F**) Male genitalia, internal sac incomplete. (**G**–**I**,**g**–**i**) Tegmen. (**J**–**L**,**j**–**l**) Median lobe. (**A**,**a**,**C**,**D**,**G**,**g**,**J**,**j**) Ventral views. (**B**,**b**,**F**,**I**,**i**,**L**,**l**) Dorsal views. (**E**,**H**,**h**,**K**,**k**) Lateral views. Scale bar: 2 mm.

Figure 15A–F and Figure 16A–L,a,b,g–l.

Chinese common name: 齿跗锯天牛.

*Prionus sifanicus* Plavilstshikov, 1934: 220 [10]. Type locality: China: Chongqing (Giu-Fu Shan).

*Prionus sifanicus*: Gressitt, 1951: 24, 27 [12]; Hua, 2002: 226 (catalogue) [14]; Drumont & Komiya, 2006: 4, Figures 19, 20 [22]; Danilevsky, 2009: 690 [23]; Löbl & Smetana, 2010: 94 (catalogue) [15]; Li et al., 2014: 93 [16]; Chen et al., 2019: 23 (catalogue) ([5], part, wrong type locality); Lazarev, 2019: 1213 [24]; Danilevsky, 2020: 116 (catalogue) [17]; Drumont & Komiya, 2021: 130, Figure 4 [6].

**Complementary description.** Body length 18.0–28.5 mm, humeral width 8.2–12.0 mm.

**Male genitalia** (Figure 16A–L,a,b,g–l): Tergite VIII (Figure 16B,b) transverse, apex broad and slightly emarginated in middle, setae very short and moderately dense. Tegmen rhombic in ventral view (Figure 16G,g), slightly curved in lateral view (Figure 16H,h); parameres (Figure 16D) narrower apically, moderately slender (length/width ca. 2.0) with rounded apex; entirely covered with dense short setae. Median lobe very slightly curved (Figure 16K,k) in lateral view, median struts subequal to 4/7 of median lobe. Ventral plate of median lobe strongly projected (Figure 16D,J,j), dorsal plate of median lobe with two wide lobes (Figure 16L,l). Internal sac with two pieces of crest-shaped basal armature (Figure 16J–L,j–l).

Female unknown.

**Examined material:** Lectotype ♂ (Figure 15A,B) specify, Szechuan, Giu-Fu Shan (重庆金佛山), 1500–2000 m, [E.] Reitter (ZMUM).

**China, Chongqing:** 2♂♂ (Figure 16C–F), Chongqing, Jiangjin (重庆江津), 10.VII.1981, leg. Qi-Zhen Wang (IZCAS).

**Distribution:** China (Chongqing).

**Remarks:** The distribution records from Sichuan were all based on the type locality Jinfoshan. Before 1997, Chongqing was a part of Sichuan Province; therefore, all the previous authors wrote Szechuan [10,23,24], Sichuan [14,22], or Chongqing, Sichuan [5,6,16,17]. The two additional males examined in this study were from Jiangjin, which belongs to Chongqing, though the labels marked “Sichuan, Jiangjin” (四川江津). Up to now, no specimens from Sichuan Province, as defined today, i.e., after the separation of Chongqing, have been reported.

## 4. Discussion

**Figure 17:**
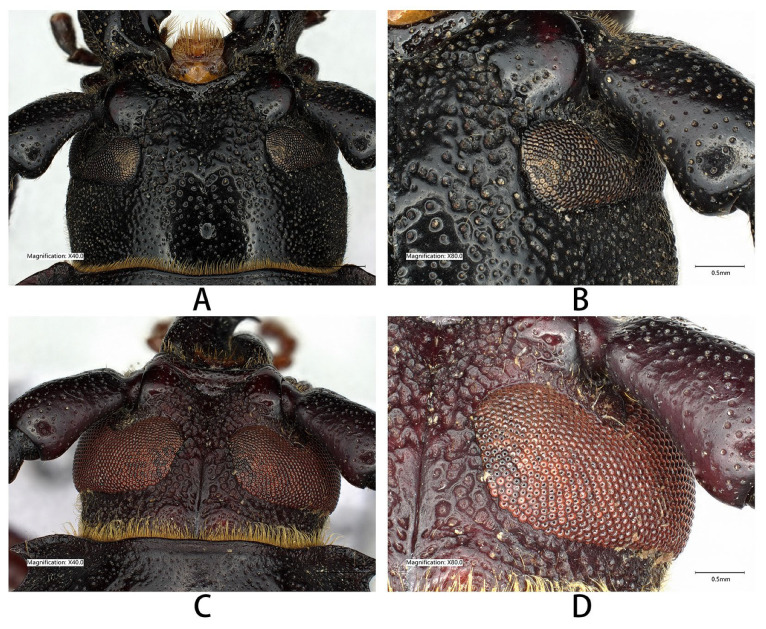
Compound eyes of *Prionus* spp. (**A**,**B**) *Prionus gahani* Lameere, 1912, male from Chongqing, same specimen as Figure 10C. (**C**,**D**) *Prionus delavayi delavayi* Fairmaire, 1887, male from Guangdong. Scale bars: 1 mm for A, C and 0.5 mm for B, D.

No biological information of the “*Prionus gahani*” species group was available before this study. Only rare type specimens were known for all the five previously known species [3,6,8,9,10]. Based on the collecting experiences from Ming-Yu Zhu, who collected two males of *P. zhumingyui* sp. nov, Cheng-Zhi Bian, who offered four males of *P. sontinh* from Yunnan, Tao-Kun Liao and Liang Guo, who collected a series of *P. antonkozlovi*, and Hao Xu and his students, who collected quite many males of *P. gahani* from Sichuan, we concluded that the species of this group are not coming to the light and are active during the day. This does not match the previous knowledge about Prioninae: “Adults are typically crepuscular or nocturnal and of somber colors; brightly colored (sometimes mimetic) or metallic diurnal species are few and mostly tropical” [25]. The species of this group are diurnal, although they have somber colors. Their small compound eyes (Figure 17A,B) (especially compared with other *Prionus* species, which have large compound eyes (Figure 17C,D) and are attracted to light) are well-matched to their diurnal lifestyle. As members of Prioninae, the adults appear to be relatively short-lived and do not feed, and this might be one of the reasons for their relative rarity.

## 5. Actualized List of Species Composing the “*Prionus gahani*” Species Group

*Prionus antonkozlovi* Drumont & Komiya, 2021

*Prionus gahani* Lameere, 1912

*Prionus lameerei* Semenov, 1927

*Prionus sifanicus* Plavilstshikov, 1934

*Prionus sontinh* Do, Drumont & Komiya, 2019

*Prionus zhumingyui* Lin & Drumont, **sp. nov.**

## Data Availability

The original contributions presented in this study are included in the article. Further inquiries can be directed to the corresponding author.
